# miR-31 Functions as an Oncomir Which Promotes Epithelial-Mesenchymal Transition via Regulating BAP1 in Cervical Cancer

**DOI:** 10.1155/2017/6361420

**Published:** 2017-10-11

**Authors:** Nan Wang, Yong Li, Jianhong Zhou

**Affiliations:** ^1^Laboratory of Molecular Biology, College of Life Science, Jiaying University, Meizhou 514015, China; ^2^Key Laboratory of Molecular Biophysics of the Ministry of Education, College of Life Science and Technology, Center for Human Genome Research, Huazhong University of Science and Technology, Wuhan 430074, China

## Abstract

MicroRNA-31 (miR-31) functions as tumor suppressors or oncogenes that are involved in tumor behavior. However, the function of miR-31 in cervical carcinogenesis remains unclear. The aim of this study was to validate the potential role of miR-31 and BRCA1-associated protein-1 (BAP1) on regulating epithelial-mesenchymal transition (EMT) in cervical cancer. In the present study, qRT-PCR assay revealed that the expression of miR-31 was upregulated in human cervical cancer cells and clinical tissues. Results of wound healing and cell migration assay revealed that knockdown of miR-31 inhibited cell metastasis and migration. Bioinformatic and dual-luciferase reporter gene assay showed that BAP1 was the direct target of miR-31. Furthermore, the results revealed that miR-31 promoted proliferation and EMT in cervical cancer cells and accelerated the development of tumor growth* in vivo* xenograft experiment by inhibiting BAP1 expression. Overall, these results highlight an important role of miR-31 functioning as an oncomir which could promote EMT in cervical cancer via downregulating BAP1 expression. Thus, downregulation of miR-31 could be a novel approach for the molecular treatment of cervical cancers and other malignancies.

## 1. Introduction

Cervical cancer, a common malignancy in gynecology, is the fourth leading cause of cancer-related deaths in female worldwide [1]. Although the major risk factor causing cervical cancer is persistent infection with high-risk human papillomaviruses (HPVs), accumulating evidences have shown that HPVs infection alone is insufficient to cause the malignant transformation and there may be other genetic alterations in cervical carcinogenesis [2]. Even though the overall survival rate of cervical cancer cases was increased owing to surgery, chemotherapy, and/or radiotherapy, the prognosis of cervical cancer patients remains poor. Metastasis and invasion are the main causes of death in cervical cancer cases; however, the molecular mechanisms of metastasis and invasion in cervical carcinogenesis are still poorly understood. Finding novel metastases-related genes and exploring their molecular mechanism are of great significance.

MicroRNAs (miRNAs) are a class of single-stranded, noncoding RNAs (~22 nucleotides in length), which binds to the 3′ untranslated region (3′UTR) of target gene, leading to mRNA degradation or translational repression [3, 4]. Recent evidences recognized that aberrant miRNAs expression is associated with cell malignant transformation, especially in tumorigenesis and tumor development [5, 6]. The specific expression of miRNAs in each cancer type may serve as a biomarker for cancer diagnosis and prognosis or even as therapeutic target. miRNAs function as oncogenes or tumor suppressors that can suppress multiple tumor suppressor genes or oncogenes during carcinogenesis [7]. Among these miRNAs, miR-31 may function as an oncogene or a tumor suppressor depending on the cellular contexts [8–11]. To date, miR-31 has been reported as upregulated in cervical cancer, which implies that miR-31 may serve as an onco-miRNA participating in the development of cervical cancer [12]. Mounting investigations have indicated the essential role of epithelial-mesenchymal transition (EMT) in the progression of human cancers [13–15]. EMT is under the control of miRNAs network [16]. Whether miR-31 can induce EMT or other malignant transformation in cervical cancer remains unknown.

BRCA1-associated protein-1 (BAP1) is a deubiquitinating enzyme, which has been reported to correlate with numerous cellular processes. Overexpression of BAP1 can suppress tumor growth in mouse xenografts [17]. In NSCLC, high BAP1 expression was associated with a lack of lymph node metastasis [18]. Yu et al. found that BAP1 was a target of miR-31 and inhibited lung cancer progression [19]. However, there has very little research on the role of BAP1 in cervical cancer and the relationship between miR-31-BAP1 and EMT has not yet been reported.

In this study, we first identified that miR-31 expression is inversely correlated to the expression of BAP1. BAP1 was a direct target of miR-31; downregulation of BAP1 by miR-31-induced EMT in cervical cancer. Therefore, our study clarified previously unidentified prometastatic roles of miR-31 in cervical cancer and miR-31-BAP1 pathway might be a new potential target for therapy in cervical metastasis.

## 2. Materials and Methods

### 2.1. Patient Samples and Cell Culture

Cervical cancer samples were collected from Medical College of Jiaying University, China. Both of the carcinomas and normal adjacent tissues were collected. The clinicopathologic information of the patients is listed in Table 1. This study was approved by local institutional review boards on human subject research and in accordance with the Declaration of Helsinki. Written informed consent was obtained from all study participants.

The human cervical cancer cell lines HeLa, HaCaT, and C33A were obtained from Chinese Center for Type Culture Collection (Shanghai, China) and grown in Dulbecco's modified Eagle's medium supplemented with 10% fetal bovine serum (FBS; Gibco Life Technologies) at 37°C in a humidified 5% CO_2_/95% air atmosphere.

### 2.2. MicroRNA Transfection

The human cervical cancer cells were seeded at 6-well plate and transfected with miR-31, miR-31 negative control (miR-NC), miR-31 inhibitor (anti-miR-31), or miR-31 inhibitor negative control (anti-miR-NC) using Lipofectamine 2000 (Invitrogen) on the following day when the cells were approximately 60–70% confluent, following the manufacturer's instructions. The miRNAs were purchased from RiboBio (RiboBio Co., Guangzhou, China). The full length human BAP1 plasmid and empty plasmid which served as the negative control were purchased from Invitrogen. The cells were harvested 48 h after transfection and subjected to analysis by quantitative RT-PCR or Western blot. The sequences of BAP1 used were listed as follows: (si-BAP1#1: 5′-CCGUGAUUGAUGAUGAUAUTT-3′ (sense); si- BAP1#2: 5′-CGGCCUUUCUAGACAAUCATT-3′(sense); and si-BAP1#3: 5′-GGCUGAGAUUGCAAACUAUTT-3′ (sense). Primer sequences of miR-31 were as follows: miR-31-p-F CCGGACGCGTGCACAAAAGTTATACATAATGTCATTATTCTTATG

miR-31-p-R GCCCAAGCTTCAGTTCCAAGTTACAGGAGAATACTATGA.

### 2.3. RNA Extraction and RT-PCR

Total RNA was extracted from cells using Trizol reagent (Invitrogen Life Technologies). The concentrations of RNA were determined using a NanoDrop ND-1000 instrument (ThermoFisher, Waltham, MA, USA) and aliquots of the samples were stored at −80°C. The real-time PCR was performed as reported previously [12].

### 2.4. Protein Extraction and Western Blot Analysis

All cells were rinsed with PBS and lysed with RIPA Lysis buffer (Beyotime, China) supplemented with a Protease and Phosphatase Inhibitor Cocktail (Thermo Scientific 78440) on ice for 30 min. Cell lysates were centrifuged for 10 min (12000*g*, 4°C), the precipitation was collected, and then total protein concentration was determined using BCA Protein Assay Kit (Beyotime, China). Proteins were separated by 12% SDS-PAGE and then transferred to polyvinylidene fluoride (PVDF) membrane (Millipore, Boston, MA, USA). The membranes were blocked in 5% nonfat milk for 2 h at room temperature and incubated with the primary anti-BAP1, anti-E-cadherin, anti-N-cadherin, anti-vimentin antibody, and anti-GAPDH antibody. The membranes were washed for three times and then incubated with secondary antibody for 2 h at room temperature. The signals were detected with ECL Western Blotting Substrate (ThermoFisher, Waltham, MA, USA) and protein band intensity was measured using Quantity One software. Three independent experiments were performed.

### 2.5. Luciferase Activity Assay

To detect BAP1 as the direct binding target of miR-31, a luciferase reporter assay was performed. HeLa cells were cultured in 24-well plate and cotransfected with miR-31 and BAP1 3′-UTR wild type or mutation of the putative miR-31 target region using Lipofectamine 2000 (Invitrogen). Forty-eight hours after transfection, luciferase activity was measured with dual-luciferase reporter assay system (Promega, USA). Luciferase activity was normalized to corresponding Renilla luciferase activity. All experiments were performed in three independent times.

### 2.6. Immunohistochemical Stain

Immunohistochemical (IHC) staining assay was performed according to our previously published protocols [12]. In brief, formaldehyde-fixed, paraffin-embedded tissue sections were dewaxed in xylene solution and rehydrated using graded ethanol solutions; antigen-retrieval was performed using citrate buffer at 90°C for 30 minutes. Then, the slides were incubated in primary antibody at 4°C overnight, and horseradish peroxidase-conjugated secondary antibody was then added. The colour reaction was developed with 330-diaminobenzidine tetrahydrochloride/0.03% H_2_O_2_, followed by counterstaining with hematoxylin.

### 2.7. Cell Proliferation Assay

The cervical cancer cells (5 × 10^3^ per well) were seeded in 96-well plates and transfected on the following day. Cell proliferation was determined at 24, 48, 72, and 96 h using the CellTiter 96 AQueous One Solution Cell Proliferation Assay Kit (Promega), according to the manufacturer's instructions as we previously described [12]. The assays were performed in triplicate and were repeated three times.

### 2.8. Wound Healing Assay

Cells were seeded in 6-well plate and cultured until 90%–100% confluent in complete medium. Cell monolayers were wound using a 200 *μ*l pipette tip through the monolayer, and then the layer was washed with PBS to remove cell debris. Photographic images were captured from the same region at 0 and 48 h for each wound.

### 2.9. Transwell Migration Assay

Cell invasion assay was using Matrigel-coated Transwell chamber (8 *μ*m pore size; Corning, USA). 5 × 10^4^ cells/well transfected with miR-31 or miR-NC were seeded into the upper chamber of 24-well Transwell plates containing 1% FBS medium. After 48 h, the upper chamber cells will migrate toward 10% FBS medium in the bottom chamber. The upper filter was mechanically removed, and the lower filter was fixed and stained with Hema-Diff Solution (Fisher Scientific, USA). Images were captured using microscope and average migrating cells from six independent fields were counted.

### 2.10. *In Vivo* Xenografts

Four-week-old female BALB/c nude mice were purchased from Shanghai SLAC Laboratory Animal, Co., Ltd. Mice were divided into two groups (*n* = 5); 5 × 10^6^ HeLa cells in which BAP1 was overexpression were subcutaneously injected into the right flank of the mice. Tumor xenograft diameters were measured by digital calipers every 5 days, and the tumor volume was calculated by the formula: Volume = (width)^2^ × length/2. At day 35, the mice were sacrificed and tumors were excised, fixed in formalin, and processed for further analysis. All Animals experiments were approved by the Jiaying University Animal Care and Use Committee.

### 2.11. Statistical Analysis

All data were obtained from at least three independent experiments and were presented as means ± SD. Student's *t*-test was used to analyse the differences of means between two different groups. Data were analyzed using GraphPad Prism 6.0. Statistical significance was set at *p* < 0.05.

## 3. Results

### 3.1. miR-31 Is Upregulated and Correlated with Poor Survival in Cervical Cancer

**Figure 1:**
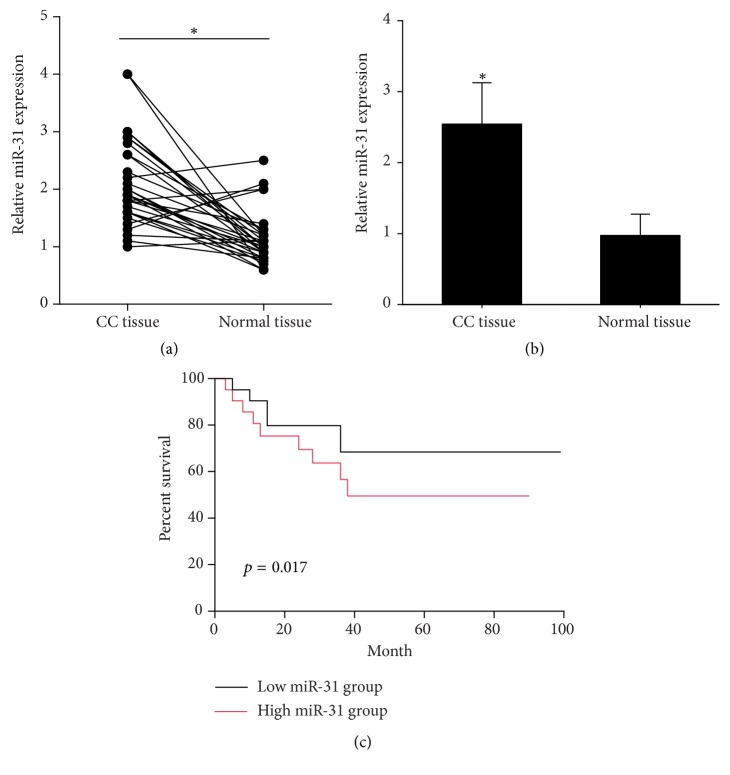
miR-31 is upregulated in cervical cancer patients and is correlated with poor prognosis. ((a) and (b)) RT-PCR analysis of the miR-31 level in 31 pairs of cervical cancer tissues and adjacent normal tissues. The expression of miR-31 was significantly upregulated in cervical cancer tissues compared with the normal tissues (*p* < 0.05). (c) Kaplan-Meier curves of the overall survival of 31 pairs of cervical cancer patients (low miR-31 group, *n* = 31; high miR-31 group, *n* = 31). Cervical cancer patients with high miR-31 expression had shorter overall survival (long-rank test, *p* < 0.05).

We use quantitative real-time PCR to confirm the altered expression level of miR-31 in cervical cancer tissues and adjacent nontumor tissues. In agreement with the previous studies, miR-31 expression was detected significantly upregulated in cervical cancer tissues compared with nontumor tissues (Figures 1(a) and 1(b)). Kaplan-Meier analysis revealed that patients with high miR-31 had less overall survival times than those with low miR-31 expression (Figure 1(c)). Furthermore, the clinicopathological parameters showed that increased expression of miR-31 was correlated with node metastasis, deep stromal invasion, vascular involvement, and FIGUREO stage and tumor size, but there was no significant correlation between miR-31 level and age and tumor size (Table 1). Taken together, these results indicate that miR-31 was involved in the progression and invasion/metastasis of cervical cancer and was correlated with worse prognosis.

### 3.2. Prediction of BAP1 as a Target of miR-31

**Figure 2:**
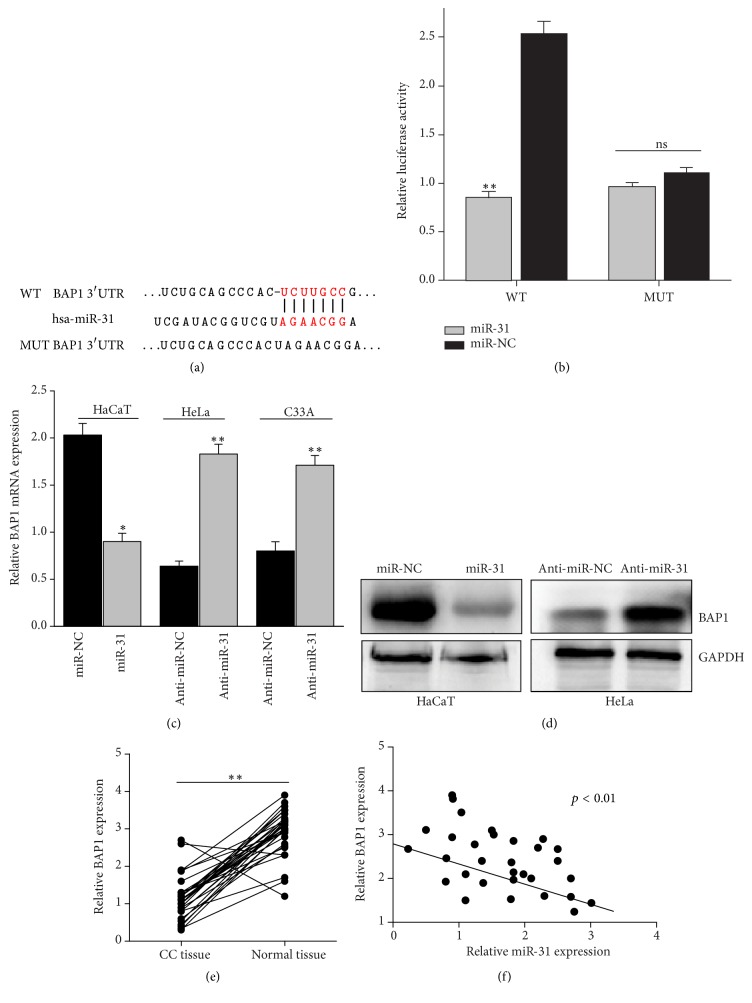
BAP1 is a direct downstream target of miR-31. (a) Putative miR-31 binding site of human BAP13′-UTR containing wild type (WT) and mutant (MUT). Either WT or MUT BAP1 3′-UTR was subcloned into the dual-luciferase reporter plasmid. (b) Relative luciferase activity of the BAP1 wild type and mutant BAP1 3′-UTR. ((c) and (d)) RT-PCR and Western blot assay showed the alteration of BAP1 and miR-31 in cervical cancer cells. Experiments were performed in triplicate. (e) RT-PCR results showed BAP1 expression in 31 cervical cancer tissues and adjacent normal tissues. (f) Inverse correlation between miR-31 and BAP1. ^*∗∗*^*p* < 0.01,  ^*∗*^*p* < 0.05.

In order to investigate the molecular mechanism by which miR-31 executed its function, four bioinformatics algorithms (TargetScan, miRanda, Microcosm, and PicTar) were used in combination to identify the more accurate potential targets of miR-31. Among the candidate genes, BAP1 was predicated as a target of miR-31 by all four algorithms and was selected for experimental verification. On the other hand, the downregulated expression of BAP1 was reported to have connection with carcinogenesis and EMT progression [17, 19], suggesting that BAP1 may serve as a tumor suppressor gene implicated in the development of human cancers. The 3′-UTR of BAP1 contained a high conserved binding site for miR-31 seed region as shown in Figure 2(a).

### 3.3. Confirming That BAP1 Is a Direct Target of miR-31

We further examined whether BAP1 was a direct target of miR-31 in cervical cancer cells using dual-luciferase reporter assay. By transient cotransfection of miR-31 with BAP1 3′-UTR wild type or mutation of the putative miR-31 target region into HeLa cells, the results showed that miR-31 significantly decreased activity of the luciferase reporters with WT 3′-UTR, but the activity of MUT 3′-UTR vector remained unaffected (Figure 2(b)). Next, we confirmed the inverse relationship between miR-31 and BAP1 expression in HeLa and HaCaT cells. Inhibition of miR-31 expression enhanced the level of BAP1 in the two cell lines (Figures 2(c) and 2(d)). These results indicate that miR-31 binds to BAP1 3′-UTR directly and negatively regulates BAP1 expression in cervical cancer cells. To further investigate the correlation between miR-31 and BAP1, we use qRT-PCR assay to detect the level of miR-31 and BAP1 expression in cervical cancer tissues and adjacent normal tissues. As shown in Figures 2(e) and 2(f), BAP1 was lower in cervical cancer tissues compared with adjacent normal tissues and had negative correlation with miR-31.

### 3.4. Upregulation of miR-31 and Knockdown of BAP1 Promoted Cell Migration* In Vitro*

**Figure 3:**
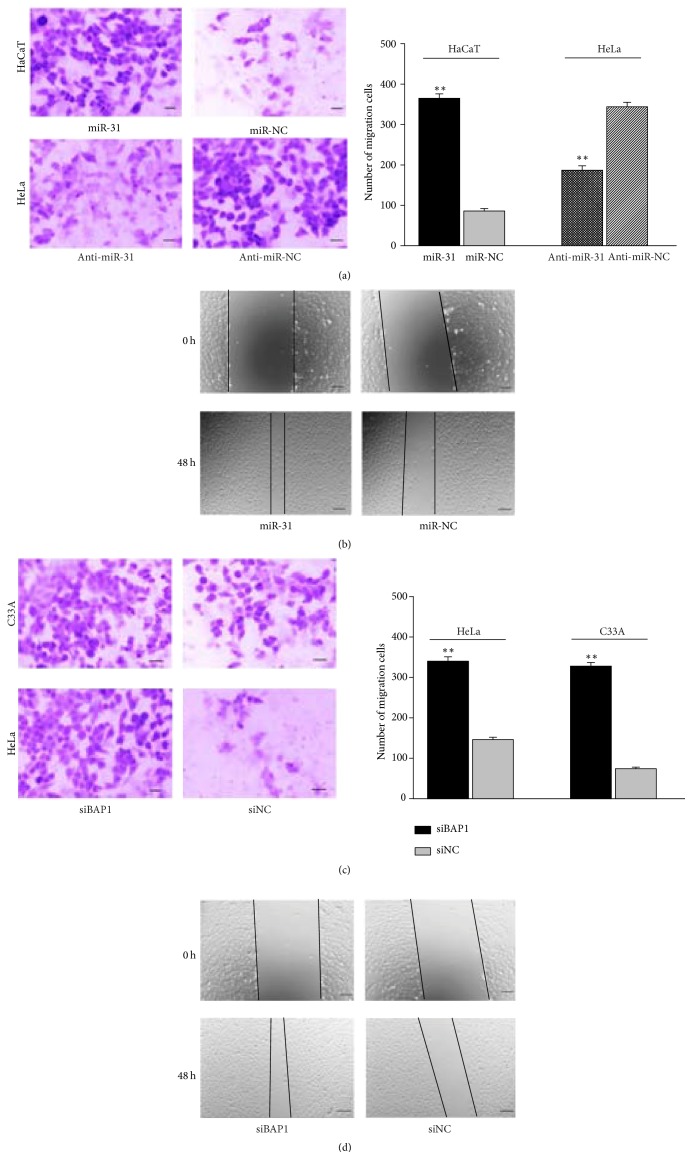
Overexpression of miR-31 and inhibition of BAP1 promoted cervical cancer migration* in vitro*. (a) Transwell migration assay was utilized to analyze the effect of miR-31/miR-31 inhibitor on the migration of HaCaT and HeLa cells. (b) Representative images of the wound healing assay using HaCaT cells transfected with miR-31/miR-NC were shown. Wound healing results in HeLa cells was not shown. (c) Representative images of Transwell migration assay using C33A and HeLa cells transfected with siBAP/siNC. (d) Analysis of the effect of siBAP on the migration of HeLa cells by wounding healing assay. The scale bar represents 50 *μ*m. Each experiment was performed in triplicate.^*∗∗*^*p* < 0.01.

To explore the role of miR-31-BAP1 in cervical cancer metastasis, Transwell migration and wound healing assay were performed in HeLa, C33A, and HaCaT cells. The results show that upregulation of miR-31 increased tumor cell migratory ability compared with the negative control cells. As expected, inhibition of miR-31 suppressed cell migration ability (Figures 3(a) and 3(b)). Knockdown of BAP1 by siRNA was also able to promote migratory ability of HeLa and C33A cells compared with the siRNA NC-treated cells (Figures 3(c) and 3(d)). Taken together, these data indicate that miR-31-BAP1 plays important role in the proliferation of cervical cancer cells.

### 3.5. The Influence of miR-31 and BAP1 on the Growth of Cervical Cancer Cells* In Vivo*

**Figure 4:**
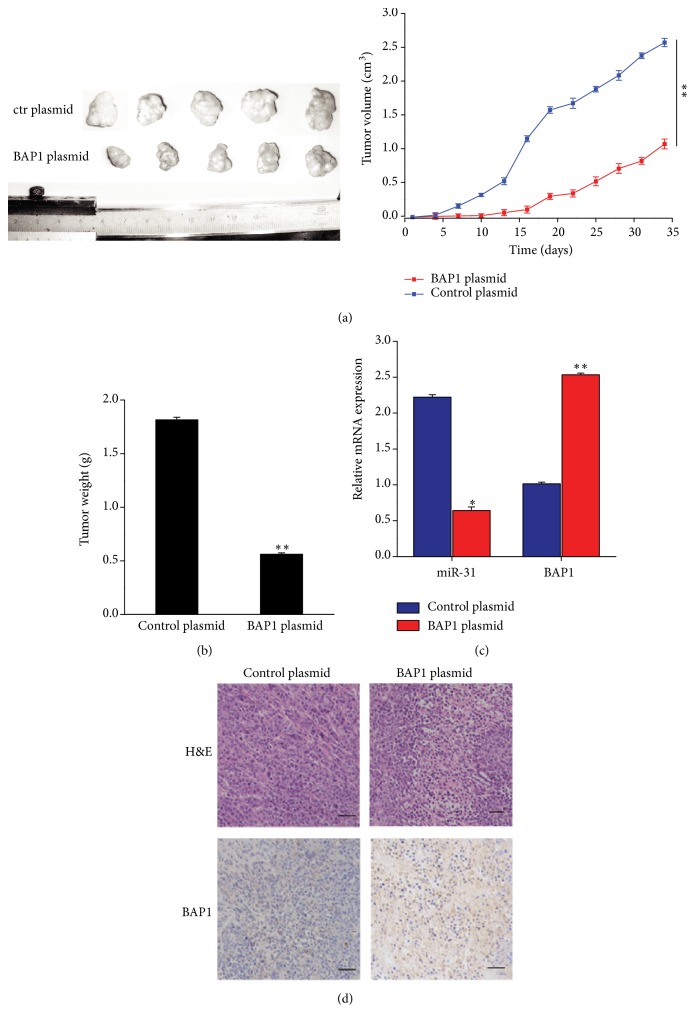
The effect of miR-31 and BAP1 on the tumorigenicity in nude mice xenograft. (a) HeLa cells were transfected with BAP1 plasmid or control plasmid. The tumors were collected on day 35 after implantation (left); tumor volume was measured at the end of the experiment (right). (b) Statistical analysis of the tumor weight. (c) Quantitative RT-PCR analysis of miR-31 and BAP1 in the tumors from different implanted mice. (d) BAP1 expression was measured by IHC in different xenograft tissues. The scale bar represents 50 *μ*m. ^*∗*^*p* < 0.05,  ^*∗∗*^*p* < 0.01.

To evaluate the effect of miR-31 and BAP1 on the growth of cervical cancer xenograft mouse model was used. As previously reported, HeLa cell was miR-31 overexpression cell line and therefore we transfected HeLa cells with a BAP1 overexpression plasmid. The HeLa cells were implanted subcutaneously into 4-week-old nude mice. After 35 days, the mice were sacrificed and the tumors were measured. BAP1 overexpression group has a significant reduction in both tumor size and weight compared with the control plasmid group (Figures 4(a) and 4(b)). The results above suggest that BAP1 overexpression reverses the effect of miR-31 on tumor growth and miR-31 could promote tumor growth by silencing BAP1 expression in cervical cancer. We also detect the mRNA and protein expression level of BAP1, tumors with BAP1 plasmid transfected group exhibited higher BAP1 mRNA and protein expression compared with the control group (Figures 4(c) and 4(d)), suggesting that BAP1 plasmid overexpression could rescue the expression of BAP1 suppressed by miR-31.

### 3.6. miR-31-BAP1 Signaling Promotes Cell Migration by Stimulating EMT

**Figure 5:**
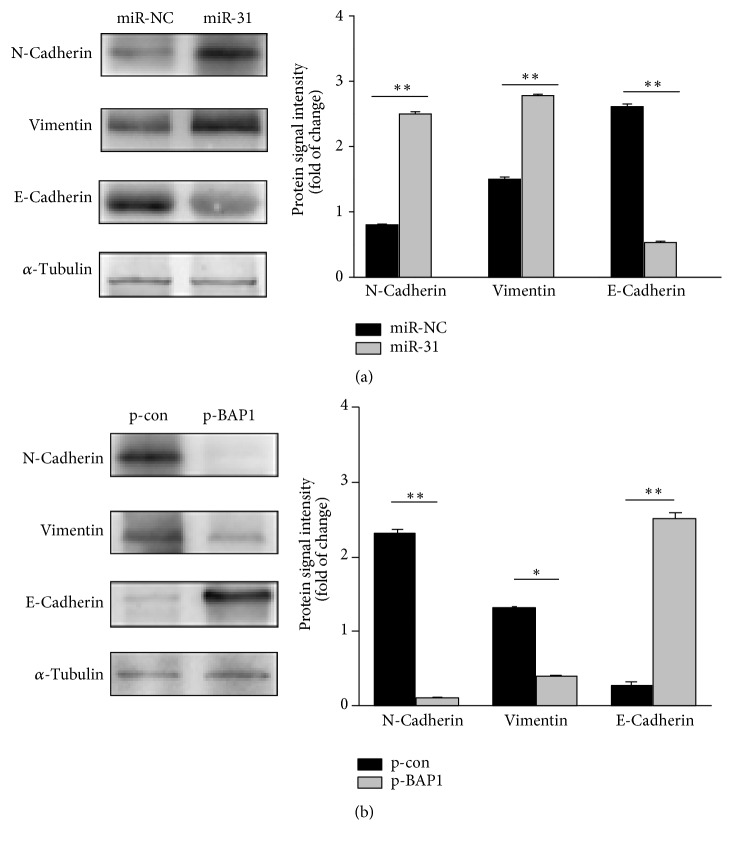
miR-31-BAP1 signal promoted tumor cell migration via stimulating EMT. Western blot analysis of the expression of EMT molecular markers, including N-cadherin, E-cadherin, and vimentin in HeLa and HaCaT cells. (a) HaCaT cells were transfected with miR-31 or miR-NC. (b) HeLa cells were transfected with BAP1 plasmid or control plasmid. Experiments were performed in triplicate.

It is well recognized that EMT is involved in tumor cell migratory and is invasive. To gain further insight into whether miR-31 was an EMT- regulatory miRNA in cervical cancer, Western blot analysis was performed to examine the EMT-related molecules in cervical cancer cells. Notably, N-cadherin and vimentin hallmarks of EMT were dramatically increased and E-cadherin, an epithelial markers, was decreased when compared to BAP1 overexpression control group (Figures 5(a) and 5(b)). These results indicated that miR-31 directly targeted BAP1 and induced EMT-like changes in cervical cancer.

### 3.7. Silencing of BAP1 Reverses the Antitumor Effects of miR-31 Inhibitor

**Figure 6:**
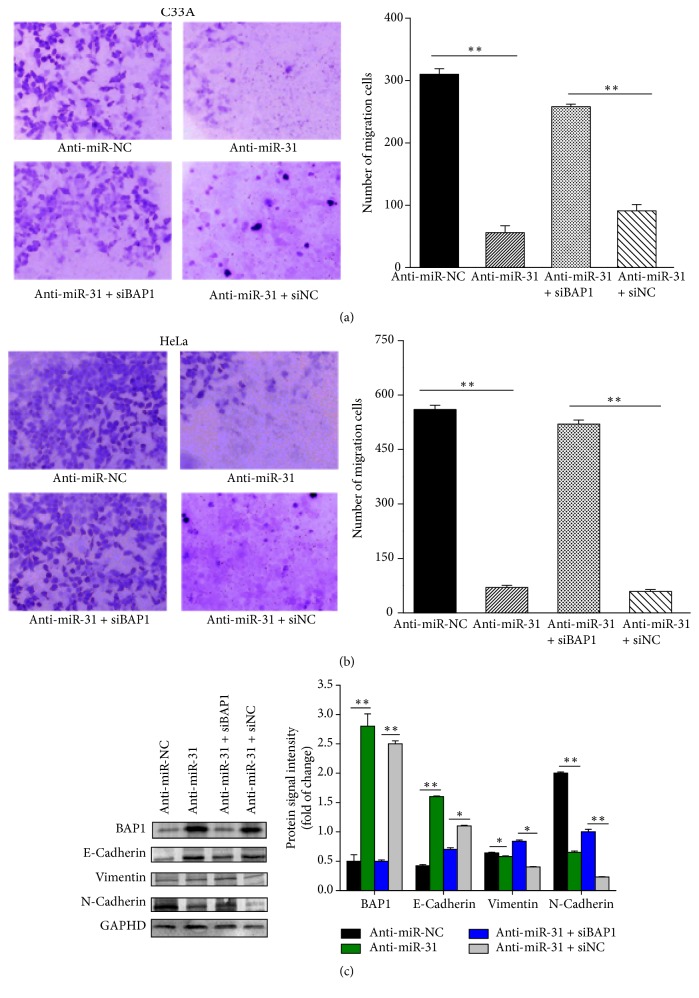
Silencing of BAP1 reverses the antitumor effects of miR-31 inhibitor. Analysis of the migratory capacity of C33A (a) and HeLa cells (b) transiently transfected with inhibition of miR-31 and cotransfected with miR-31 inhibitor and siBAP1 using Transwell assay. The mean number of migrated cells was counted from at least three random fields. ^*∗∗*^*p* < 0.01. (c) Western blot analysis of the expression of BAP1 and EMT markers in HeLa cells transiently transfected with miR-31 inhibitor and cotransfected with miR-31 inhibitor and siBAP1. The scale bar represents 50 *μ*m. ^*∗*^*p* < 0.05,  ^*∗∗*^*p* < 0.01.

To gain further insight into the regulatory effect of miR-31-BAP1 on EMT in cervical cancer, miR-31 inhibitor and BAP1 siRNA were cotransfected into HeLa and C33A cells to simultaneously silence miR-31 and BAP1 expression. We found that dual knockdown of BAP1 and miR-31 significantly reversed cell migration ability of HeLa and C33A cells induced by single-knockdown of miR-31 (Figures 6(a) and 6(b)). Moreover, downregulation of N-cadherin and vimentin and upregulation of E-cadherin by miR-31 inhibitor were also rescued by cotransfected inhibition of miR-31 and BAP1 (Figure 6(c)). Taken together, these data indicate that BAP1 is a key effector of invasion and migration in cervical cancer, which was regulated by miR-31.

## 4. Discussion

Accumulating evidences have highlighted that miRNAs participate in the tumor growth and/or metastatic process. According to the previous published papers, the role and mechanism of miR-31 in cervical cancer are mainly confined to tumor growth. In the present study, our findings indicate that miR-31 functions as an oncogenic miRNA in the progress of cervical carcinogenesis. In cervical cancer cases, upregulation of miR-31 was found to be correlated with shorter survival time and poorer prognosis. Our clinicopathological data also showed that upregulation of miR-31 was significantly associated with advanced clinical stage, tumor size, lymph node metastasis, and deep stromal invasion. This suggested that miR-31 may serve as a direct therapeutic target for cervical cancer patients. miR-31 has also been found to behave as an oncogenic miRNA in other human cancers, including lung cancer [20], colon cancer [21], head and neck cancer [22], and hepatocellular carcinoma [9]. It needs a lot of work to further investigate the mechanism of miR-31 upregulated in cervical cancer.

Recent reports suggest that aberrant expression of miR-31 plays crucial role in human cancer development, and miR-31 served as an oncogene in lung and colorectal cancer [20, 21]. Conversely, miR-31 may also act as a tumor suppressor in breast cancer [23], bladder cancer [10], and prostate cancer [11]. Thus, miR-31 functions as an oncogene or tumor suppressor dependent on the cellular types and contexts. Due to the complex effect of miR-31, our study aimed to identify its potential biological function in cervical cancer.* In vivo* and* in vitro *results revealed that the oncogenic miR-31 inhibitor inhibits the tumor growth and cell metastasis. Moreover, we further investigate the mechanism by which miR-31 enhances malignant transformation of cancer cells, and bioinformatic and dual-luciferase reporter gene assay identified the target gene for miR-31. BAP1 (BRCA1-associated protein-1), located at the chromosome region 3p21.1, is a nuclear-localized deubiquitinating enzyme that may be involved in regulation of tumorigenesis, cell cycle and growth, cell death, and DNA damage response [24]. Previous reports suggested that overexpression of BAP1 in lung cancer cells inhibited tumor growth in mice, and BAP1 has few mutations in lung cancer, suggesting that there may be another pathway involved in the dysregulation of BAP1 [17, 25, 26]. In this study, we showed that BAP1 was a direct target of miR-31 and an inverse correlation between miR-31 and BAP1 expression in cervical cancer cells and tissues. We also validated that BAP1 could promote cervical carcinogenesis and EMT progression was regulated by miR-31. BAP1 may function as a tumor suppressor in cervical cancer; it is very significant to inhibit miR-31 to induce BAP1 expression. Whether other miRNAs and signal pathways such as TGF-*β* will regulate BAP1 expression needs further investigation.

EMT endows tumor cells with higher invasive/metastatic capacities, stem cell-like characteristics, resistance to apoptosis, and immune tolerance [14]. Mounting evidences have highlighted the important role of miRNAs in EMT process. For instance, miR-489 modulated chemoresistance through EMT-related pathway in breast cancer [27]. miR-216a and miR-217-induced EMT-stimulated drug resistance via targeting PTEN in hepatocellular carcinoma [28]. Our study showed that miR-31 induced EMT via regulating BAP1 expression in cervical cancer cells and marked the expression of E-cadherin downregulated and N-cadherin and vimentin upregulated. Xenograft tumor experiment observed the effect of miR-31 on EMT, which indicated that overexpression BAP1 plasmid inhibits the tumor growth. The expressions of E-cadherin and BAP1 were increased, while the expressions of N-cadherin and vimentin were decreased compared with the control group.

In summary, we demonstrated that miR-31 can induce EMT by downregulating BAP1 in cervical cancer, and miR-31-induced acceleration of cellar migration and invasion was partially by its regulation of BAP1* in vitro* and* in vivo*. In general, miR-31 interference with BAP1 recovery expression may be a potential therapeutic strategy for metastatic cervical cancer patients.

## Figures and Tables

**Table 1:** Correlation of miR-31 expression level and clinicopathological data.

Clinicopathological parameters	miR-31 expression		*p* value
	Low (*n* = 31)	High (*n* = 31)	
Age (years)			
≤40	13	16	
>40	18	15	0.475
Tumor size			
≤5 cm	17	15	
>5 cm	14	16	0.753
Lymph node metastasis			
No	19	10	
Yes	12	21	0.007^*∗*^
FIGO stage			
IB	22	8	
IIA	9	23	0.000^*∗*^
Vascular invasion			
No	24	14	
Yes	7	17	0.000^*∗*^
Stromal invasion			
≤2/3	25	5	
>2/3	6	26	0.000^*∗*^
Differentiation			
Well	15	12	
Poor	16	19	0.174
Peritoneal metastasis			
No	18	10	
Yes	13	21	0.128
Histological type			
Serous	14	23	
Others	17	8	0.027^*∗*^

^*∗*^ *p* < 0.05.

## Abbreviations

miR-31: MicroRNA-31
BAP1: BRCA1-associated protein-1
EMT: Epithelial-mesenchymal transition
HPVs: High-risk human papillomaviruses
3′UTR: 3′ untranslated region
WT: Wild type
MUT: Mutant type
PVDF: Polyvinylidene fluoride
IHC: Immunohistochemical
qRT-PCR: Quantitative reverse transcriptional PCR.
